# Mediator subunit Med12 contributes to the maintenance of neural stem cell identity

**DOI:** 10.1186/s12861-016-0114-0

**Published:** 2016-05-17

**Authors:** Nam Hee Kim, Carolina B. Livi, P. Renee Yew, Thomas G. Boyer

**Affiliations:** Department of Molecular Medicine and Institute of Biotechnology, The University of Texas Health Science Center at San Antonio, San Antonio, TX 78229-3900 USA; Agilent Technologies, Portland, OR 97224-7154 USA

**Keywords:** Med12, Mediator, Neural stem cell, Cell adhesion, Cell cycle, Gene expression, Microarray

## Abstract

**Background:**

The RNA polymerase II transcriptional Mediator subunit Med12 is broadly implicated in vertebrate brain development, and genetic variation in human MED12 is associated with X-linked intellectual disability and neuropsychiatric disorders. Although prior studies have begun to elaborate the functional contribution of Med12 within key neurodevelopmental pathways, a more complete description of Med12 function in the developing nervous system, including the specific biological networks and cellular processes under its regulatory influence, remains to be established. Herein, we sought to clarify the global contribution of Med12 to neural stem cell (NSC) biology through unbiased transcriptome profiling of mouse embryonic stem (ES) cell-derived NSCs following RNAi-mediated Med12 depletion.

**Results:**

A total of 240 genes (177 up, 73 down) were differentially expressed in Med12-knockdown versus control mouse NS-5 (mNS-5) NSCs. Gene set enrichment analysis revealed Med12 to be prominently linked with “cell-to-cell interaction” and “cell cycle” networks, and subsequent functional studies confirmed these associations. Targeted depletion of Med12 led to enhanced NSC adhesion and upregulation of cell adhesion genes, including *Syndecan 2* (*Sdc2*). Concomitant depletion of both Sdc2 and Med12 reversed enhanced cell adhesion triggered by Med12 knockdown alone, confirming that Med12 negatively regulates NSC cell adhesion by suppressing the expression of cell adhesion molecules. Med12-mediated suppression of NSC adhesion is a dynamically regulated process in vitro, enforced in self-renewing NSCs and alleviated during the course of neuronal differentiation. Accordingly, Med12 depletion enhanced adhesion and prolonged survival of mNS-5 NSCs induced to differentiate on gelatin, effects that were bypassed completely by growth on laminin. On the other hand, Med12 depletion in mNS-5 NSCs led to reduced expression of G1/S phase cell cycle regulators and a concordant G1/S phase cell cycle block without evidence of apoptosis, resulting in a severe proliferation defect.

**Conclusions:**

Med12 contributes to the maintenance of NSC identity through a functionally bipartite role in suppression and activation of gene expression programs dedicated to cell adhesion and G1/S phase cell cycle progression, respectively. Med12 may thus contribute to the regulatory apparatus that controls the balance between NSC self-renewal and differentiation, with important implications for MED12-linked neurodevelopmental disorders.

**Electronic supplementary material:**

The online version of this article (doi:10.1186/s12861-016-0114-0) contains supplementary material, which is available to authorized users.

## Background

Development of the mammalian brain is an intricate and protracted process that initiates with neurulation in the gastrulating embryo and extends postnatally to structural and experiential maturation in the adult. This process involves a highly orchestrated and spatiotemporally restricted series of stages, involving initial neurogenesis followed by neuronal migration, differentiation, synaptogenesis, and establishment of neural connectivity [1–3]. In parallel, non-neuronal programs, including gliogenesis, myelination, and angiogenesis complete the development and maturation processes [4–6]. During neurogenesis, an initial pool of primary neural stem cells (NSCs), corresponding to neural tube-derived neuroepithelial cells, undergoes a gradual switch from symmetrical autoreplicative divisions to asymmetrical neurogenic divisions to produce a progressively restricted set of neural progenitors that, in turn, specify the final complement of neuronal subtypes and macroglia that populate individual brain structures [2, 7, 8]. An appropriate balance between symmetric (self-renewing) and asymmetric (differentiative) cell divisions is critical to ensure maintenance of an adequate pool of founder progenitors as well as the proper number and distribution of their more fate-restricted derivatives, all of which contribute to the final neuronal output. The choice between self-renewal and differentiation is largely determined by programmed gene expression changes, many at the transcriptional level, in response to signals propagated by autocrine, paracrine, and exocrine soluble factors, as well as cell-cell and cell-matrix interactions [9–12]. Accordingly, genetic or environmental perturbations that disrupt physiologic transcription controls can alter NSC fate leading to neurodevelopmental defects.

In metazoans, signal-dependent developmental regulation of RNA polymerase II (Pol II) transcription requires Mediator, a conserved multi-subunit signal processor through which regulatory information conveyed by gene-specific transcription factors is transduced to Pol II. Functionally, Mediator acts to control and coordinate multiple steps in the transcription process, including pre-initiation complex (PIC) formation through chromatin reconfiguration and Pol II recruitment, early initiation events linked to Pol II promoter escape, Pol II pausing and elongation, and co-transcriptional RNA processing [13–18]. Structurally, Mediator is assembled from multiple constituent subunits (30 in mammals) arranged into 4 distinct modules, including “head”, “middle”, “tail”, and a dissociable 4-subunit “kinase” module comprising Med13, Med12, Cyclin C (CycC), and Cdk8 (or its mutually exclusive paralog Cdk19) [19–23]. Within the kinase module, Med12 is a critical transducer of regulatory information conveyed by signal-activated transcription factors linked to diverse pathways critical for proper brain development and function, including the Sonic hedgehog, Wnt, Notch, and EGF pathways, among others [24–28]. Notably, Med12 is an obligate activator of CycC-dependent Cdk8/19 in Mediator, and Med12-mediated signaling can therefore occur in a manner dependent or independent of Cdk8/19 [13, 28–31].

Because of its critical role as an endpoint in key developmental signaling pathways, Med12 is broadly implicated in vertebrate neural development. In zebrafish, Med12 has been shown to be required for proper development of the brain and neural crest where it plays an essential role in the production of monoaminergic neurons and cranial sensory ganglia through selective regulation of neuronal-specific gene expression [32–34]. More recently, Med12 was shown to be required for hindbrain boundary formation and a notable reduction of cell proliferation was found in the hindbrain of Med12 mutant zebrafish [35]. Studies in hypomorphic Med12 mutant mouse embryos revealed that Med12 is essential for early mouse development and required for neural tube closure and axis elongation, which is likely to be caused by the disruption of the Wnt/β-catenin and Wnt/planar cell polarity (PCP) signaling [36]. Finally, in humans, genetic variation in MED12 is associated with cognitive and behavioral dysfunction. For example, polymorphisms in the MED12 C-terminus have been linked with neuropsychiatric illness, including schizophrenia and psychosis [37–39]. Furthermore, missense mutations in MED12, located on Xq13, have been shown to be causative for a growing number of X-linked intellectual disability (XLID) disorders, including FG (or Opitz-Kaveggia), Lujan-Fryns (or Lujan), and Ohdo (MKB type) syndromes [40–42]. Recent molecular genetic and biochemical studies have uncovered key developmental signaling pathways dysregulated as a consequence of these pathogenic mutations in MED12, suggesting possible bases to explain at least some of the clinical phenotypes associated with these developmental disorders. In this regard, MED12 mutations linked with FG, Lujan and Ohdo syndrome have all been shown to disrupt epigenetic repression of neuronal gene expression imposed by the RE1 silencing transcription factor/neural restrictive silencer factor (REST/NRSF), a master regulator of neuronal fate [13, 29, 42]. Furthermore, MED12 mutations linked with FG and Lujan syndromes were shown to disrupt a Mediator imposed constraint on GLI3-dependent SHH signaling [27, 28]. Nonetheless, a more complete description of Med12 function in the developing nervous system, including the specific biological networks and cellular processes under its regulatory influence, remains to be firmly established. With this goal in mind, we herein combined targeted Med12 depletion with global transcriptome profiling in mouse NSCs (mNSCs). These studies reveal a functionally bipartite role for Med12 in the concurrent suppression and activation of gene expression programs dedicated to cell adhesion and G1/S phase cell cycle progression, respectively, thus revealing an unexpected role for Med12 in the enforcement of two genetic programs critical to maintenance of the NSC state.

## Results

### Med12 regulates NSC gene expression programs linked to adhesion and cell cycle progression

To investigate the global contribution of Med12 to NSC biology, we profiled the transcriptomes of mouse NS-5 (mNS-5) NSCs following RNAi-mediated Med12 depletion. The mNS-5 cell line is a clonal embryonic stem (ES) cell-derived adherent neural stem cell line that is self-renewing, genetically stable, and multipotent with the capacity to differentiate into neurons and glia both in vitro and in vivo [43, 44]. mNS-5 NSCs were employed for transcriptome profiling in an effort to facilitate identification of target genes likely to mediate the function of Med12 in neural development. To this end, parallel cultures of mNS-5 NSCs were infected with lentiviruses expressing either non-silencing control or Med12-specific shRNAs. We validated efficient Med12 knockdown (40–60 %) at both the mRNA and protein levels by RT-qPCR and immunoblot analyses, respectively (Additional file 1: Figure S1A and B). Biotin-labeled cRNA probes synthesized from control and Med12 knockdown cells (*n* = 3) were hybridized onto Illumina MouseWG-6 BeadChip microarrays containing 45,281 unique transcript probes corresponding to >20,000 mouse genes. Genes with a False Discovery Rate (FDR)-adjusted *p*-value of *p* ≤ 0.05 and fold change ≥2 were considered differentially expressed upon MED12 depletion.

Using these criteria, a total of 240 genes were differentially expressed upon Med12 depletion in mNS-5 NSCs (Additional file 2: Table S1); 177 genes (74 %) were upregulated, while 63 genes (26 %) were downregulated. To validate primary data derived from microarray analysis, we profiled the expression levels of 29 randomly selected genes (25 upregulated, 4 downregulated) using the same RNA samples subjected to microarray analysis as well as RNA from an independent set of knockdown experiments (Additional file 1: Figure S1C and D). The results of this analysis were fully concordant with the microarray data regarding the direction and extent of regulation for each gene.

To gain insight concerning the possible biological functions of Med12 in NSCs, we subjected the list of genes differentially expressed in Med12 knockdown cells to Ingenuity® Pathway Analysis (IPA®). The most prominent molecular networks and cellular functions to emerge from this analysis included “cell-to-cell signaling and interaction”, “gene expression”, and “cell cycle” (Table 1). In addition, these genes are prominently linked to “cancer” and “genetic disorders” (including “psychological disorders”) (Table 1), consistent with the established roles of MED12 as a both cancer driver and a XLID gene [13, 39, 45–47]. While the generic association between Med12 and “gene expression” revealed by IPA is fully consistent with the well-characterized role of Med12 as a coregulator of signal-controlled transcription factors, the association of Med12 with “cell-to-cell interaction” and “cell cycle” networks, two genetic programs critical to the NSC phenotype, was unexpected. We therefore sought to explore the involvement of MED12 in these two cellular processes.

**Table 1:** Top networks and biological functions of Med12 regulated genes in mNSC

Top networks		Score
Cell-to-cell signaling and interaction, Connective tissue development and function, Cellular movement		48
Gene expression, Cell cycle, Connective tissue development and function		38
Dermatological disease and condition, Genetic disorder, Gene expression		27
Cell-to-cell signaling and interaction, Cardiac Necrosis/Cell Death, Cell death		26
Psychological disorders, Connective tissue development and function, Gene expression		22
Disease and disorders	*p*-value	# Genes
Cancer	3.02E-05–2.03E-02	56
Genetic disorder	1.93E-04–1.88E-02	95
Endocrine system disorders	2.17E-04–1.83E-02	39
Metabolic disease	2.17E-04–1.83E-02	39
Hematological disease	8.26E-04–1.83E-02	3
Molecular and cellular functions	*p*-value	# Genes
Cell-to-cell signaling and interaction	3.49E-07–1.88E-02	23
Gene expression	2.60E-05–2.03E-02	15
Cell cycle	6.34E-05–1.95E-02	13
Cellular development	8.42E-05–2.03E-02	39
Cellular movement	1.03E-04–1.98E-02	29

Ingenuity® Pathway Analysis (IPA®) was used to analyze microarray data to gain insight of putative function of Med12 in mNSC. The significance of representation (*p*-value) is determined by IPA based on the number of genes found in each biological category divided by the number of known genes assigned to that category by the Ingenuity® Knowledge Base

### Med12 suppresses NSC adhesion by repressing the expression of cell adhesion molecules

**Fig. 1:**
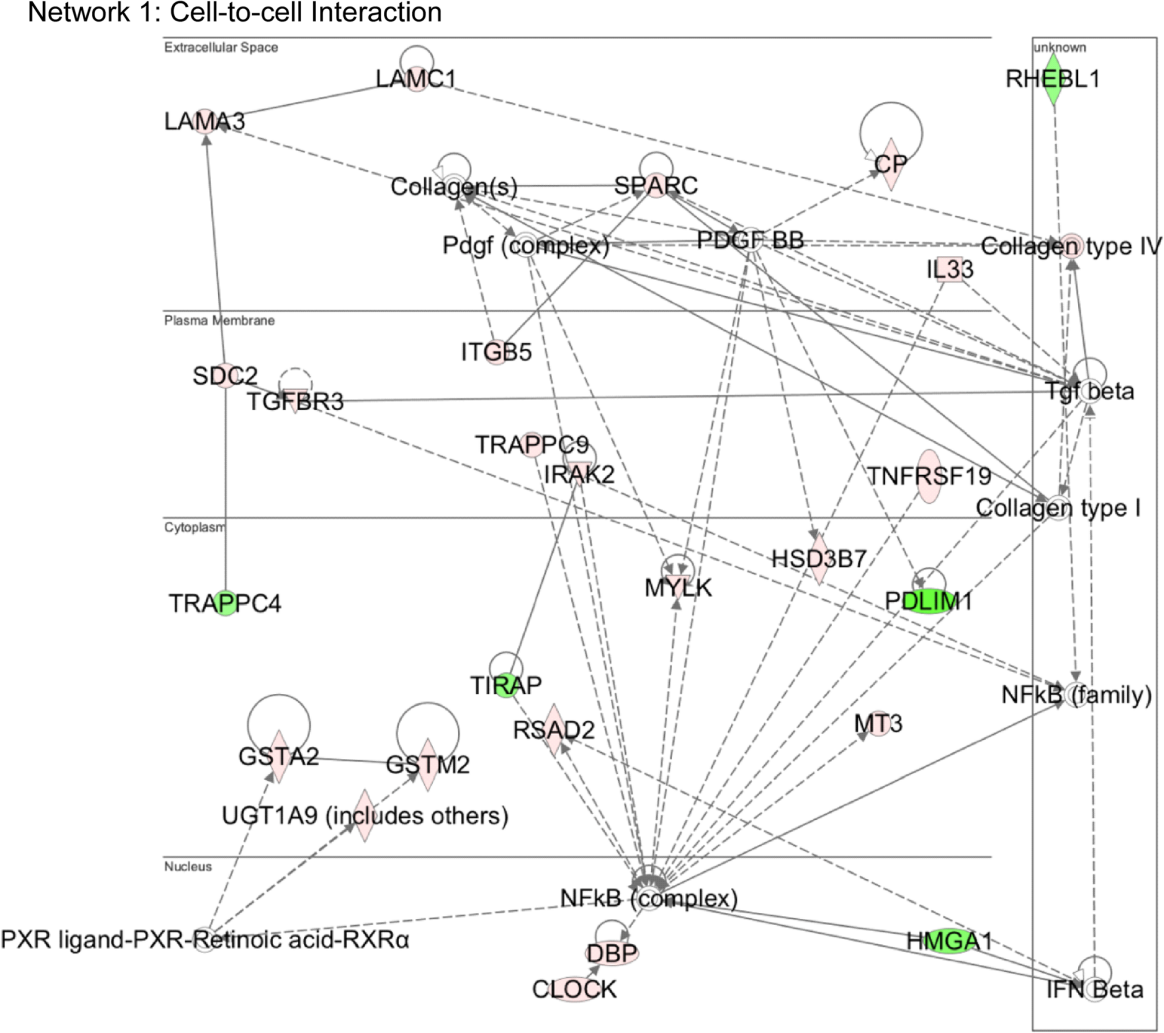
Med12 mediates suppression of genes involved in “Cell Adhesion”. Functional gene interaction networks were identified by IPA®. Genes are represented as nodes, and the biological relationship between two nodes is represented as a *line*. The color of the node indicates up- or down- regulation (*red*, up; *green*, down); uncolored nodes represent genes that were not identified as differentially expressed in our microarray, but have nonetheless been integrated into the computationally generated networks on the basis of the evidence stored in the IPA knowledge memory indicating a relevance to this network

**Fig. 2:**
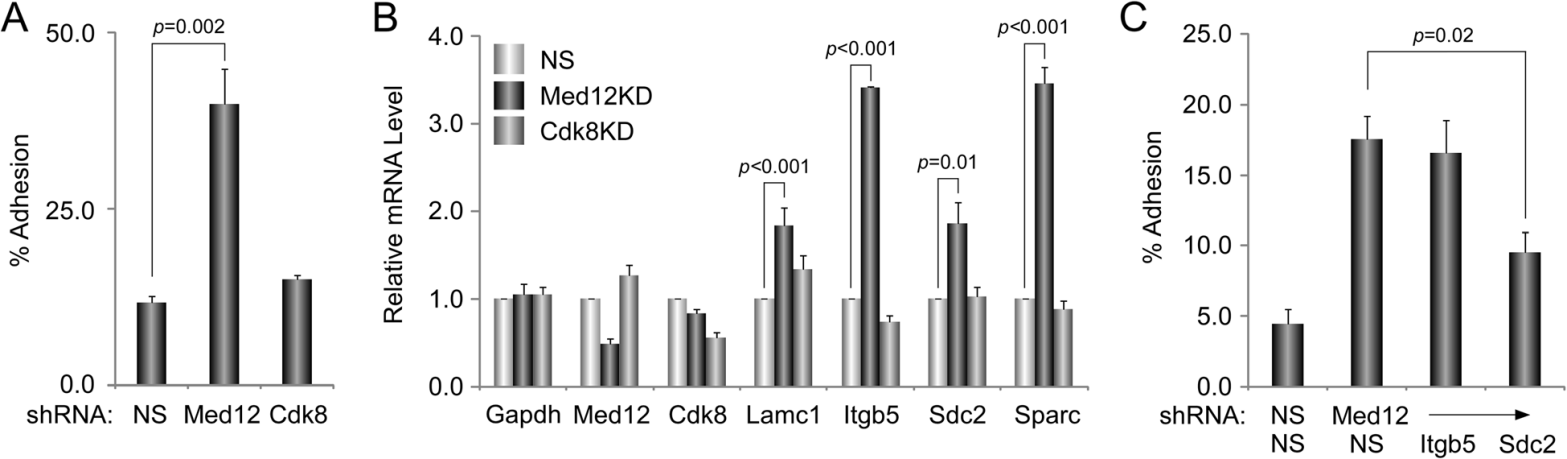
Med12 depletion enhances NSC adhesion in a manner reversible by concurrent depletion of Sdc2. **a** mNS-5 NSCs infected with lentiviruses expressing non-specific (NS) control, Med12-, or Cdk8-specific shRNAs were harvested and incubated with Calcein-AM prior to seeding onto 96-well plates. After washing, adherent cells were detected by fluorometry. The percentage of adhesion was calculated by dividing the background-subtracted fluorescence of adherent cells by the total corrected fluorescence of cells added to each well and multiplying by 100. **b** RNA from mNS-5 NSCs infected with lentiviruses expressing NS control, Med12-, or Cdk8-specific shRNAs as indicated were used for RT-qPCR, mRNA levels for each gene were normalized to β-actin mRNA and expressed relative to their corresponding mRNA levels in NS control shRNA-expressing cells. **c** mNS-5 NSCs co-infected with lentiviruses expressing shRNAs specific for Med12 or the indicated cell adhesion genes were assayed for cell adhesion as described in (**a**). 7All data represent the mean +/− SEM of at least three independent experiments performed in triplicate. *p* values were calculated by Student’s *t* test

Among Med12-regulated genes linked by IPA to the “cell-to-cell interaction” network, most were upregulated following Med12 knockdown, suggesting that Med12 might normally function to suppress a gene expression program dedicated to NSC interaction and adhesion (Fig. 1; Additional file 2: Table S1). To investigate this possibility, we monitored the impact of Med12 knockdown on the adhesive properties of mNS-5 NSCs as well as the expression levels of network genes considered likely to be direct effectors of cell adhesion, including the extracellular matrix (ECM) and/or cell membrane protein-encoding genes Laminin alpha 3 (Lama3), Laminin gamma 1 (Lamc1), Secreted acidic cysteine rich glycoprotein (Sparc), Syndecan 2 (Sdc2), and Integrin beta 5 (Itgb5). To distinguish between Cdk8-dependent and Cdk8-independent functions for Med12 in the possible regulation of cell adhesion, we additionally monitored the impact of Cdk8 knockdown on mNS-5 NSC adhesion and gene expression. To this end, mNS-5 NSCs infected with lentiviruses expressing control, Med12-, or Cdk8-specific shRNAs were comparatively evaluated using a quantitative flourometric-based cell adhesion assay (Calcein AM). We validated efficient depletion of Med12 and Cdk8 proteins in these experiments by immunoblot analysis of virally infected cell extracts (Additional file 3: Figure S2). Compared to control knockdown, Med12 knockdown enhanced mNS-5 cell adhesion by approximately 3.5 fold (Fig. 2a). By contrast, Cdk8 knockdown had no measurable impact on mNS-5 cell adhesion (Fig. 2a). As expected, we confirmed enhanced expression of cell adhesion genes (Lamc1, Itgb5, Sdc2, Sparc) upon Med12 depletion, while Cdk8 knockdown was without effect (Fig. 2b). These results validate both microarray and IPA analyses and, more importantly, implicate Med12 in the regulation of NSC adhesion, likely through Cdk8-independent suppression of a cell adhesion gene expression program.

To confirm this possibility, we asked whether enhanced mNS-5 cell adhesion observed upon Med12 depletion could be functionally reversed by concurrent depletion of cell adhesion molecules regulated by Med12. Accordingly, mNS-5 cells were co-infected with lentiviruses expressing control or Med12-specific shRNAs along with individual lentiviruses expressing shRNAs specific for either Itgb5 or Sdc2 prior to harvest and assay for cell adhesion. Strikingly, concomitant depletion of both Sdc2 and Med12 effectively reversed enhanced cell adhesion triggered by Med12 knockdown alone, thus confirming that Med12 regulates NSC adhesive properties by suppression of cell adhesion genes (Fig. 2c).

**Fig. 3:**
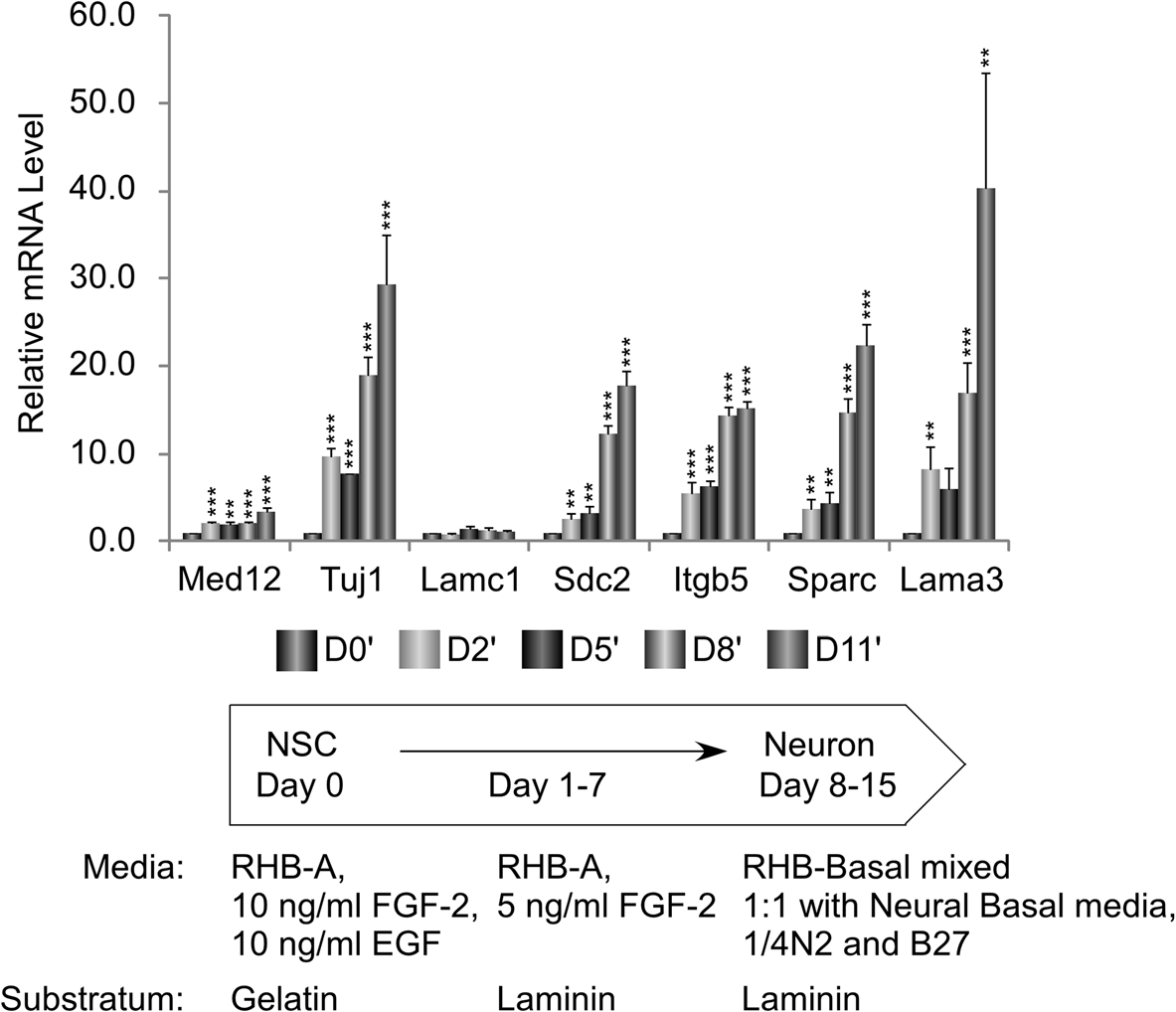
Expression of Med12-regulated cell adhesion genes increases during neuronal differentiation of mNS-5 NSCs. mNS-5 NSCs were seeded onto laminin-coated plates prior to initiation of neuronal differentiation by sequential withdrawal of growth factors as indicated in the schematic and described in Methods. RNA isolated from cells on 0, 2, 5, 8, and 11 days after initiation of neuronal differentiation was subjected to RT-qPCR. mRNA levels for each gene were normalized to β-actin mRNA and expressed relative to their corresponding mRNA levels on day 0 (D0′) of the differentiation protocol. Data represent the mean +/− SEM of three independent experiments performed in triplicate. *Asterisks* denote statistically significant differences in the relative mRNA levels for each gene compared to their corresponding levels on D0′ (Student’s *t* test, **p < 0.05, ***p < 0.01)

mNS-5 NSCs are multipotent adherent neural stem cells capable of self-renewal in the presence of growth factors, including EGF and FGF-2, and growth on gelatin. This cell line can be directed to differentiate along the neuronal lineage by sequential removal of growth factors as well as a change in substratum from gelatin to laminin that reflects the involvement of cell-cell and cell-matrix interactions in the neuronal differentiation process [43]. We sought to determine whether Med12-imposed suppression of cell adhesion genes in self-renewing NSCs cells is subject to regulation during neuronal differentiation. To this end, we first investigated whether cell adhesion genes actively repressed by Med12 in proliferating mNS-5 cells undergo changes in their respective expression levels during in vitro neuronal differentiation. For this purpose, mNS-5 cells were seeded onto laminin-coated plates and induced to differentiate along the neuronal lineage by sequential withdrawal of growth factors from the culture medium. RNAs were harvested on Day 0, 2, 5, 8, and 11 following initiation of neuronal differentiation, and the expression levels of cell adhesion genes were monitored by RT-qPCR. Strikingly, four out of five analyzed cell adhesion genes actively suppressed by Med12 in proliferating mNS-5 NSCs, including Sdc2, Itgb5, Sparc, and Lama3, were dramatically upregulated during the course of neuronal differentiation, which was confirmed by expression of the neuronal marker Tuj1 (Fig. 3). A minimal increase in Lamc1 expression, while reproducibly observed during neuronal differentiation, nonetheless failed to achieve statistical significance. Notably, the expression level of Med12 itself was significantly, albeit minimally, upregulated during neuronal differentiation. This observation excludes the possibility that neurogenic expression of Med12-targeted cell adhesion genes derives from extinction of Med12 expression during differentiation, and instead indicates dynamic regulation of Med12-mediated suppression. Apparently, alleviation of a Med12-imposed block to the expression of cell adhesion genes in self-renewing NSCs is required for, or consequent to, NSC cell differentiation.

**Fig. 4:**
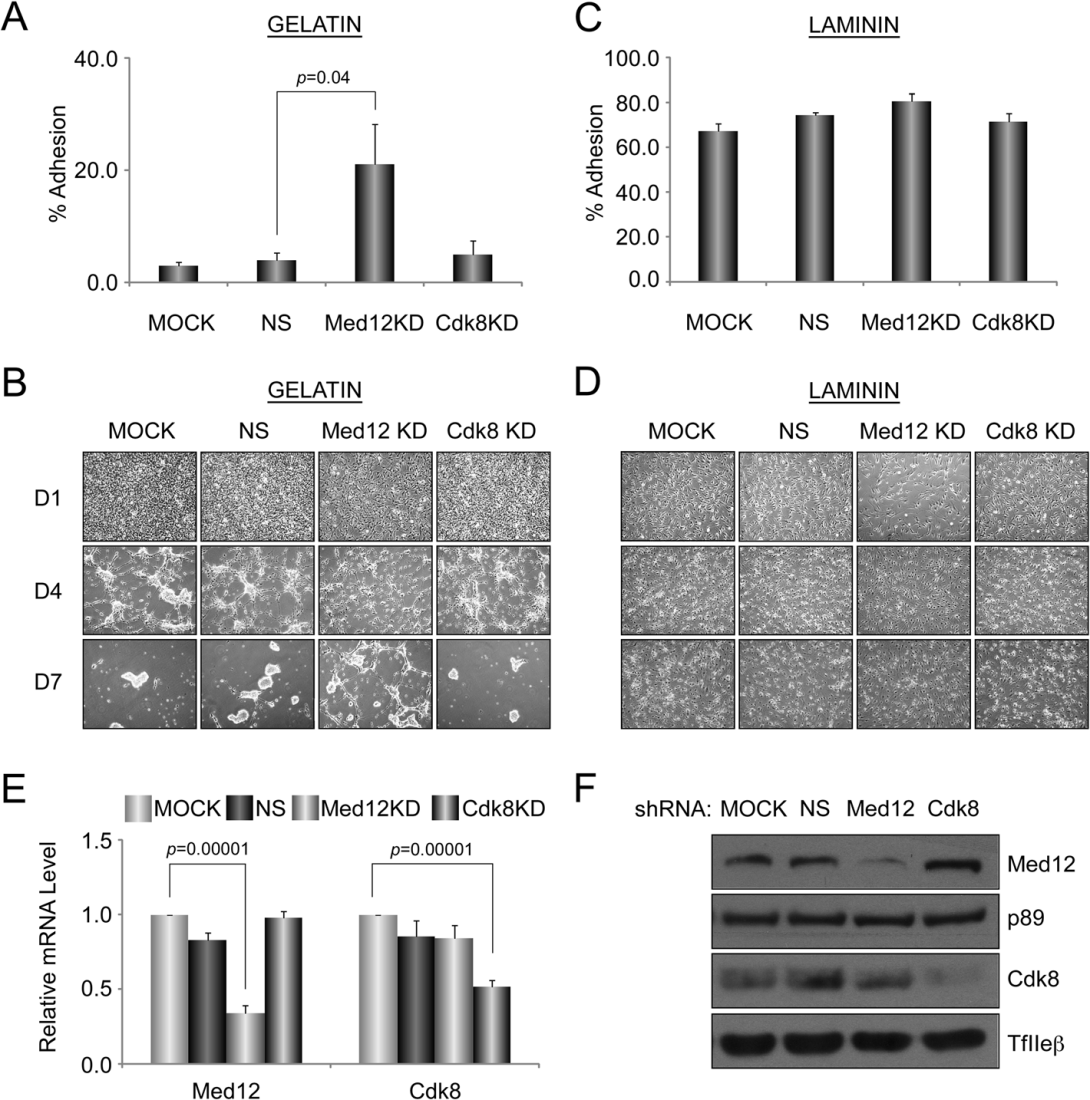
Med12 knockdown increases cell adhesion and preserves cell viability on limiting (gelatin) substratum during neuronal differentiation. **a**-**d** mNS-5 NSCs infected with lentiviruses expressing NS control, Med12-, or Cdk8-specific shRNAs were seeded into T-75 tissue culture flasks coated with gelatin (**a**, **b**) or laminin (**c**, **d**) prior to initiation of neuronal differentiation as described in Fig. 3. Fluorescence based adhesion assay (**a**, **c**) was performed 40 h after initiation of the neuronal differentiation. Data represent the mean +/− SEM of at least three independent experiments performed in triplicate. *p* values were calculated by Student’s *t* test. Brightfield images (**b**, **d**) were obtained by optical microscopy at 1, 4, and 7 days after initiation of neuronal differentiation. **e** and **f** Validation of Med12 and Cdk8 depletion in knockdown cells by RT-qPCR (**e**) and immunoblot (**f**) analyses. mRNA levels for each gene in (**e**) were normalized to β-actin mRNA and expressed relative to their corresponding mRNA levels in untreated (MOCK) cells. Data represent the mean +/− SEM of at least three independent experiments performed in triplicate. *p* values were calculated by Student’s *t* test

To begin to clarify this issue, we asked whether Med12 depletion would permit the promiscuous differentiation of NSCs on a limiting (gelatin) substratum otherwise non-permissive for differentiation. To this end, mNS-5 NSCs infected without (mock) or with lentiviruses expressing control, Med12-, or Cdk8-specific shRNAs were plated onto gelatin or laminin substrata prior to induction of neuronal differentiation by sequential withdrawal of growth factors. Changes in cell morphology and adhesion were monitored during the course of differentiation by optical microscopy and Calcein AM-based cell adhesion assay as described previously. Notably, when induced to differentiate on gelatin, Med12 knockdown cells, compared to mock-infected or control-knockdown cells, exhibited enhanced cell adhesion (~7-fold) and prolonged survival, although these cell failed to complete neuronal differentiation (Fig. 4a and b). On the other hand, enhanced cell adhesion and survival on gelatin triggered by Med12 knockdown was completely bypassed by induction of mNS-5 NSC differentiation on laminin. Thus, compared to mock-infected or control-knockdown cells, Med12-knockdown cells exhibited no significant differences in cell morphology or adhesion (Fig. 4c and d). These findings suggest that reversal of a Med12-mediated block to the expression of cell adhesion genes is a regulated process that may be required as a course of NSC differentiation along the neuronal lineage.

### Med12 promotes NSC proliferation through activation of G1/S phase cell cycle regulatory genes

**Fig. 5:**
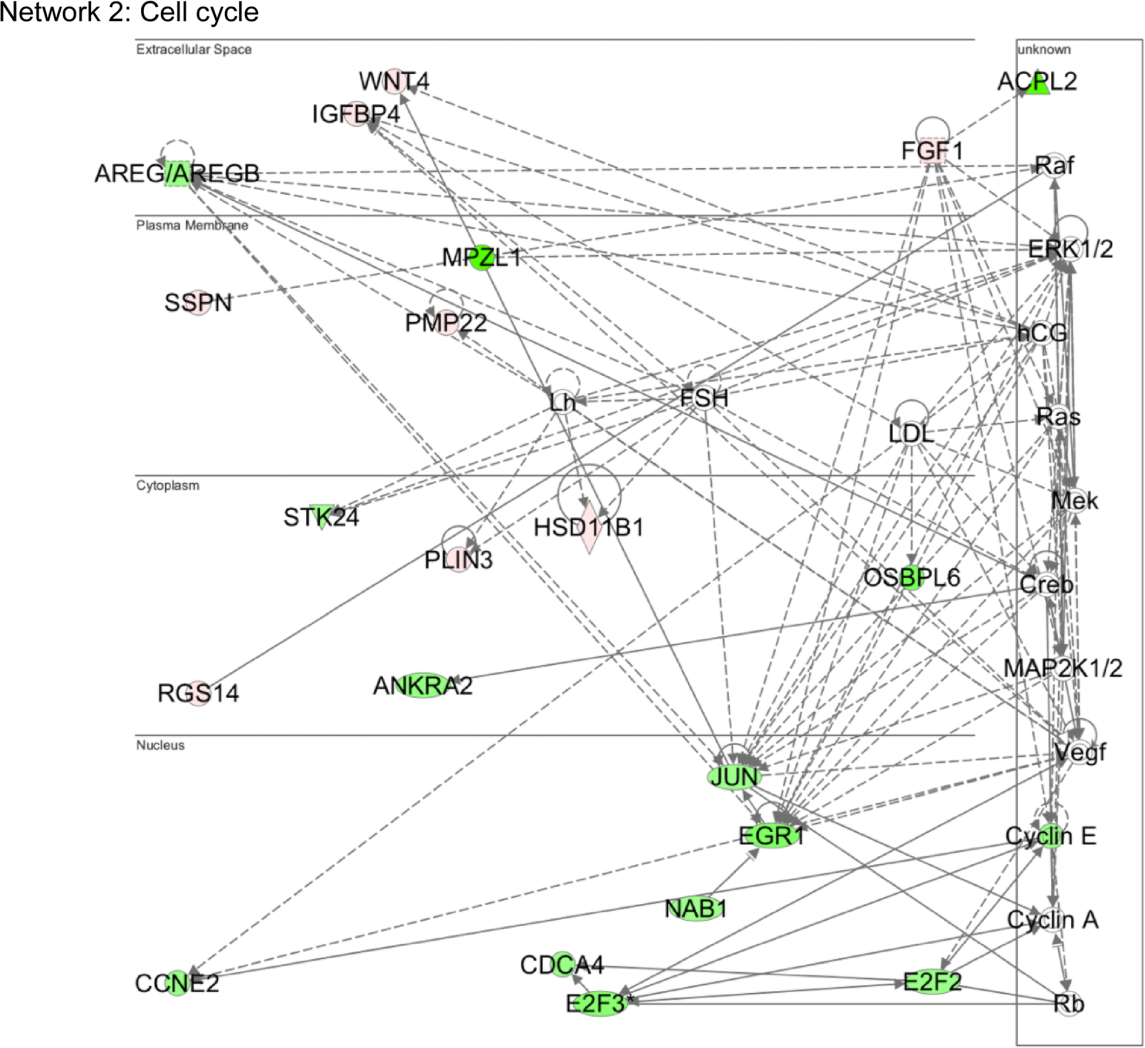
Med12 mediates activation of genes involved in “Cell Cycle Control”. Genes involved in cell cycle control are largely downregulated (*green*) upon Med12 depletion in mNS-5 NSCs. Functional gene interaction networks were identified by IPA® as described in Fig. 1

**Fig. 6:**
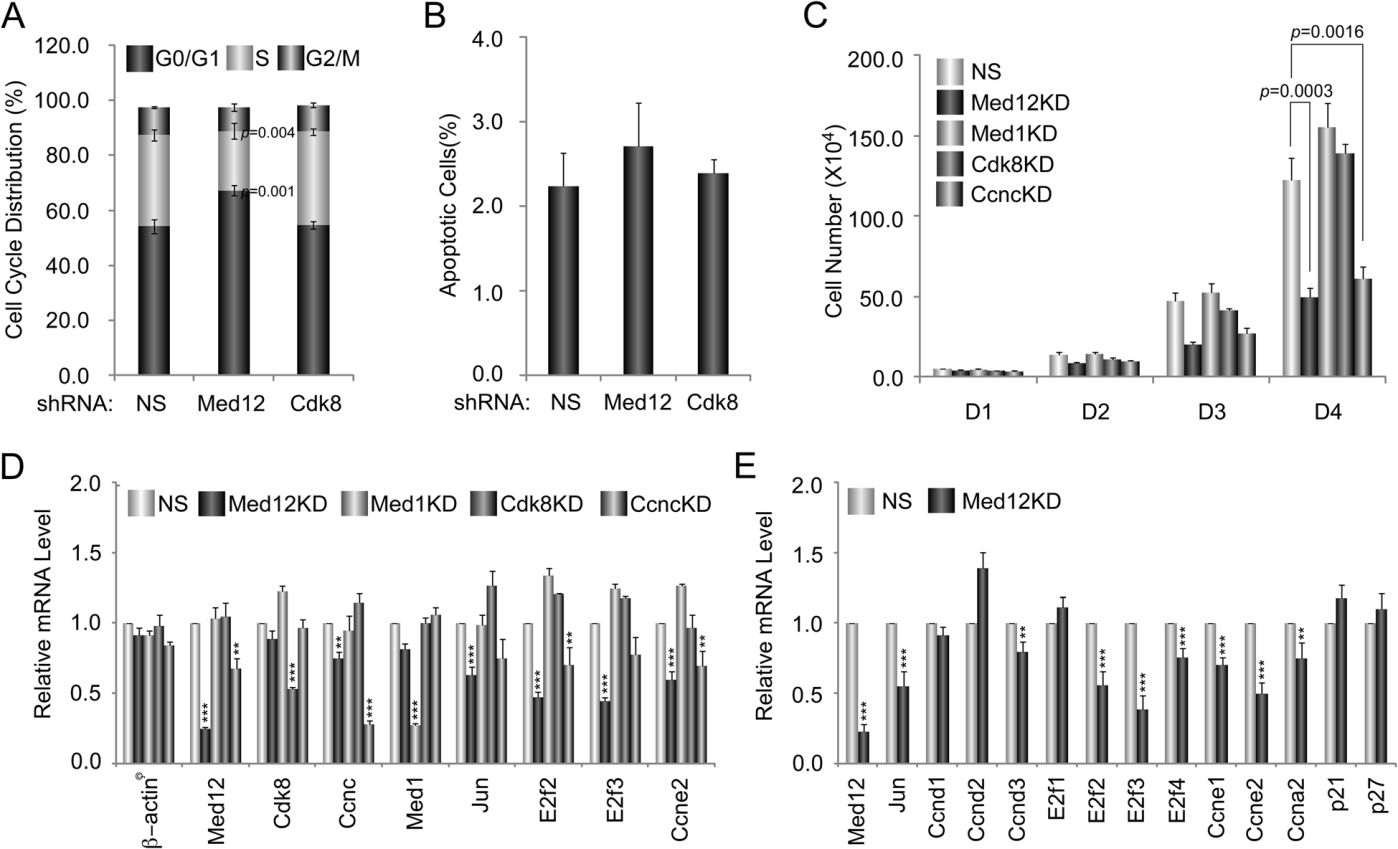
Med12 depletion reduces mNS-5 NSC proliferation likely through a G1/S phase cell cycle block. **a**-**e** mNS-5 NSCs were infected with lentiviruses expressing NS control, Med12-, Med1-, Cdk8-, and/or CycC (Ccnc)-specific shRNAs as indicated. **a** Five days post-infection, cells were harvested, fixed, and monitored for cell cycle distribution using a FACSCalibur flow cytometer and CellQuest Pro software prior to data analysis using FlowJo software. At least 10,000 single cell events were collected for the analysis. **b** Five days post-infection, cells were stained with annexin V conjugated to APC and propidium iodide. Stained cells were analyzed by FACSCalibur and data was analyzed using FlowJo software. **c** Lentivirus-infected cells were seeded at a concentration of 5 × 10^4^ cells/well in 12-well plates. Cell proliferation was monitored by counting viable cells using 0.4 % trypan blue at 24, 48, 72, and 96 h post-seeding. **d**, **e** mRNA expression levels for the indicated genes were determined by RT-qPCR analyses as described in Fig. 2. mRNA levels for each gene were normalized to Gapdh mRNA and expressed relative to their corresponding mRNA levels in control NS shRNA-expressing cells. All data represent the mean +/− SEM of at least three independent experiments performed in triplicate. *p* values were calculated by Student’s *t* test. *Asterisks* denote statistically significant differences relative to NS control shRNA (*p < 0.1, **p < 0.05, ***p < 0.01)

Among Med12-regulated genes linked by IPA to the “cell cycle”, most were downregulated following Med12 depletion (Fig. 5; Additional file 2: Table S1). Notably, several of these genes, including Ccne2, E2f2, E2f3, Jun, and Egr1, encode established G1/S phase cell cycle regulators, suggesting that in proliferating NSCs, Med12 might normally function to activate a gene expression program involved in G1/S phase cell cycle progression. To examine this possibility, we monitored the impact of Med12 knockdown on the cell cycle distribution profile of proliferating mNS-5 NSCs. Thus, asynchronously growing mNS-5 NSCs were infected with lentiviruses expressing control, Med12-, or Cdk8-specific shRNAs prior to analysis of DNA content by flow cytometry. This analysis revealed that depletion of Med12, but not Cdk8, significantly increased the percentage of G1-phase cells while simultaneously decreasing the percentage of S-phase cells, indicative of a G1/S phase cell cycle block (Fig. 6a). By contrast, Med12 depletion had no significant impact on mNS-5 apoptotic cell death (Fig. 6b). To determine if the observed G1/S phase cell cycle block accompanying Med12 knockdown is manifest in a proliferative defect, we monitored the impact of Med12 knockdown on mNS-5 proliferation by viable cell counting. Compared to control-knockdown cells, Med12-knockdown cells exhibited a significant (~2.5-fold) reduction in cell proliferation (Fig. 6c). By contrast, knockdown of neither Cdk8 nor Med1, a different subunit localized to the middle module of Mediator, adversely affected cell proliferation (Fig. 6c). Notably, however, knockdown of CycC recapitulated the impact of Med12 depletion (Fig. 6c). Because CycC links Cdk8/19 to Med12 in the Mediator kinase module, the requirement for Med12 and CycC, but not Cdk8, implies a probable role for Cdk19 in mNS-5 NSC proliferation. As expected, we confirmed significant downregulation in these experiments of G1/S phase cell cycle regulators, including Ccne2, E2f2, E2f3, and Jun, following depletion of Med12 and CycC, but not Cdk8 or Med1 (Fig. 6d). Furthermore, by targeted small-scale gene expression profiling in mNS-5 NSCs, we identified an expanded set of apparent Med12-dependent G1/S phase cell cycle regulators. Thus, Ccnd3, E2f4, Ccna2, and Ccne1 were all specifically downregulated following Med12 depletion (Fig. 6e). Notably, neither p21 nor p27 expression levels were altered by Med12 depletion, excluding DNA-damage and/or restriction checkpoints as possible bases for the G1/S phase cell cycle block observed in Med12-knockdown cells. Instead, these findings suggest that Med12 promotes NSC proliferation through Cdk8-indpendent, but possibly Cdk19-dependent, activation of a G1/S phase cell cycle regulatory gene expression program.

## Discussion

Although recent studies have begun to elaborate the involvement of Med12 in the developing vertebrate nervous system, much less in known regarding the precise functions of this important Mediator subunit at the cellular level. In an initial effort to address this knowledge gap, we herein combined Med12 knockdown with global transcriptome profiling in mouse ES cell-derived NSCs. Our findings reveal novel roles for Med12 in the control of NSC adhesion and cell cycle progression, both of which are known to be fundamentally associated with stem cell maintenance. Previous studies have indicated that interactions between NSCs and their niche play an essential regulatory role in balancing stem cell maintenance and differentiation [48, 49]. Moreover, studies have suggested that ECM, a major niche component, plays a key regulatory function in NSC maintenance and differentiation by transferring environmental cues to embryonic and adult NSCs [50–55]. For instance, in the developing mammalian embryonic brain, loss of the adhesion molecule Integrin promotes neural stem cell detachment from the ECM and ultimately apoptosis [56]. Laminins are the best-described ECM component and laminin γ1 has been shown to be associated with NSC differentiation in the mouse [57]. In addition, studies have suggested that cell adhesion molecules may influence asymmetric stem cell division by interacting with microtubules of the mitotic spindle and directing the plane of cell division [58]. Thus, while the contribution of cell adhesion molecules to NSC homeostasis is well established, it is less clear how these molecules are transcriptionally regulated. We found that Med12 depletion enhanced NSC adhesion concordant with upregulation of cell adhesion genes, including those encoding ECM components, integrins, and proteoglycans. Furthermore, concurrent reduction of both Sdc2 and Med12 was sufficient to partially reverse enhanced cell adhesion triggered by Med12 knockdown alone, confirming that Med12 negatively regulates NSC cell adhesion by transcriptional suppression of cell adhesion molecules. Notably, we observed that cell adhesion genes suppressed by Med12 in proliferating NSCs were upregulated during directed neuronal differentiation in vitro, thus revealing Med12-imposed suppression to be a dynamically regulated process. In support of this, depletion of Med12 alone was sufficient to promote enhanced adhesion and prolonged survival of NSCs induced to differentiate on a limiting (gelatin) substratum otherwise nonpermissive for neuronal differentiation. By contrast, directed differentiation of NSCs on a permissive (laminin) substratum completely bypassed the adhesion and survival advantage conferred by Med12 depletion. Taken together, our findings support the notion that Med12 contributes to maintenance of the NSC state through suppression of a cell adhesion program and, furthermore, that this suppression is alleviated as a course of neuronal differentiation.

During neural development, NSCs undergo symmetric and asymmetric divisions, the former to maintain an adequate founder progenitor pool and the latter to ensure an appropriate number and distribution of more fate-restricted progeny. Cell fate decisions are tightly associated with the cell cycle machinery [59, 60]. Cell cycle progression is controlled by checkpoints at each phase of the cell cycle by a series of highly regulated events, including the alternating activities of various cyclin-dependent kinases (CDKs). CDKs are activated only upon binding to their corresponding cyclin(s) and repressed by CDK inhibitors [61]. Previous studies have indicated that down-regulation of cyclins acting at the G1 and S phases of the cell cycle prevents S phase entry and favors withdrawal from the cell cycle [62]. Our findings revealed that Med12 depletion leads to down-regulation of G1/S cell cycle regulators, triggering a G1/S phase cell cycle block and diminished NSC proliferation. Although our results clearly indicate that Med12 plays a role in NSC proliferation, it is presently unclear whether Med12 plays a role in NSC self-renewal. In this regard, it is well established that mechanistic control of cell cycle progression is tightly linked with NSC proliferation and differentiation in the embryonic and adult nervous system [63]. For example, the G1 phase (restriction) check point, beyond which cells are irreversibly committed to replication absent external growth stimuli, represents a fundamental step in regulating stem cell self-renewal and differentiation, while G1 phase duration directly influences the differentiation of neural precursors [64–67]. Thus, Med12 may prevent unscheduled NSC differentiation by activating G1/S phase cell cycle regulators and promoting G1/S phase cell cycle transit.

Med12 is an obligate activator of CycC-dependent Cdk8 in Mediator, and Med12 may therefore control gene expression in a manner dependent or independent of Cdk8 kinase activity [13, 28, 29]. Herein, we found that Med12 regulates NSC adhesion and cell cycle regulatory genes in a Cdk8-independent manner. However, this does not exclude the possibility that Med12-mediated gene regulation in this setting is dependent on Mediator-associated kinase activity. In fact, our observation that CycC depletion effectively recapitulates the impact of Med12 knockdown supports a probable role for Med12-dependent Cdk19 activity in this context, since CycC physically links Med12 with Cdk8/19 and Cdk8 is functionally dispensable for Med12-dependent control of mNS-5 proliferation and cell cycle regulatory gene expression. Notably, Cdk19 is expressed in the developing nervous system, and we found that Cdk19 is indeed expressed in mNS-5 NSCs. Cdk8 and Cdk19 share 91 % overall amino acid sequence identity, with a particularly high degree of sequence conservation in their respective kinase and cyclin-binding domains, but considerably more divergence in their corresponding C-termini [68]. These paralogous subunits assemble into Mediator in a mutually exclusive manner, and recent studies have revealed that Cdk8-Mediator and Cdk19-Mediator are not functionally redundant during development [13, 69]. In fact, these distinct Mediator species regulate unique gene expression programs in some cellular settings [68–70]. Accordingly, further studies will be required to establish whether and how Cdk19 contributes to Med12-dependent regulation of cell adhesion and cell cycle regulatory genes in mNS-5 NSCs.

Finally, the mechanism by which Med12 differentially represses and activates cell adhesion and cell cycle regulatory programs remains to be fully clarified. In this regard, we and others have shown that Med12 can function in a context-dependent manner as either a co-activator or co-repressor of gene transcription [24, 28, 29, 71]. For example, we previously showed that MED12 is co-activator of Wnt-stimulated β-catenin; in this context, Pol II is indirectly recruited by promoter-bound LEF/β-catenin through Mediator, with which β-catenin interacts directly through its MED12 interface [24]. By contrast, we have also shown that MED12 is a co-repressor of REST/NRSF in neuronal gene silencing through a mechanism involving epigenetic repression. In this context, the MED12 interface in Mediator links promoter-bound REST/NRSF with G9a histone methyltransferase, a dominant euchromatic H3K9 dimethylase in mammalian cells [29, 72]. It thus appears likely that Med12 mediates suppression and activation of cell adhesion and cell cycle regulatory genes, respectively, through a mechanism involving differential recruitment by gene-specific regulators. In this regard, it is notable that Rest/Nrsf has previously been implicated in the regulation of an ECM gene expression program in mouse NSCs [73]. However, in this context, Rest/Nrsf appears to be required for activation of ECM genes, and therefore functionally opposed to the role of Med12 identified herein as a suppressor of ECM and cell adhesion genes. Further studies, including those in primary brain-derived NSCs, will be required to establish the precise and broader conserved regulatory mechanism(s) by which Med12 differentially functions to suppress and activate cell adhesion and cell cycle control programs in NSCs.

## Conclusions

**Fig. 7:**
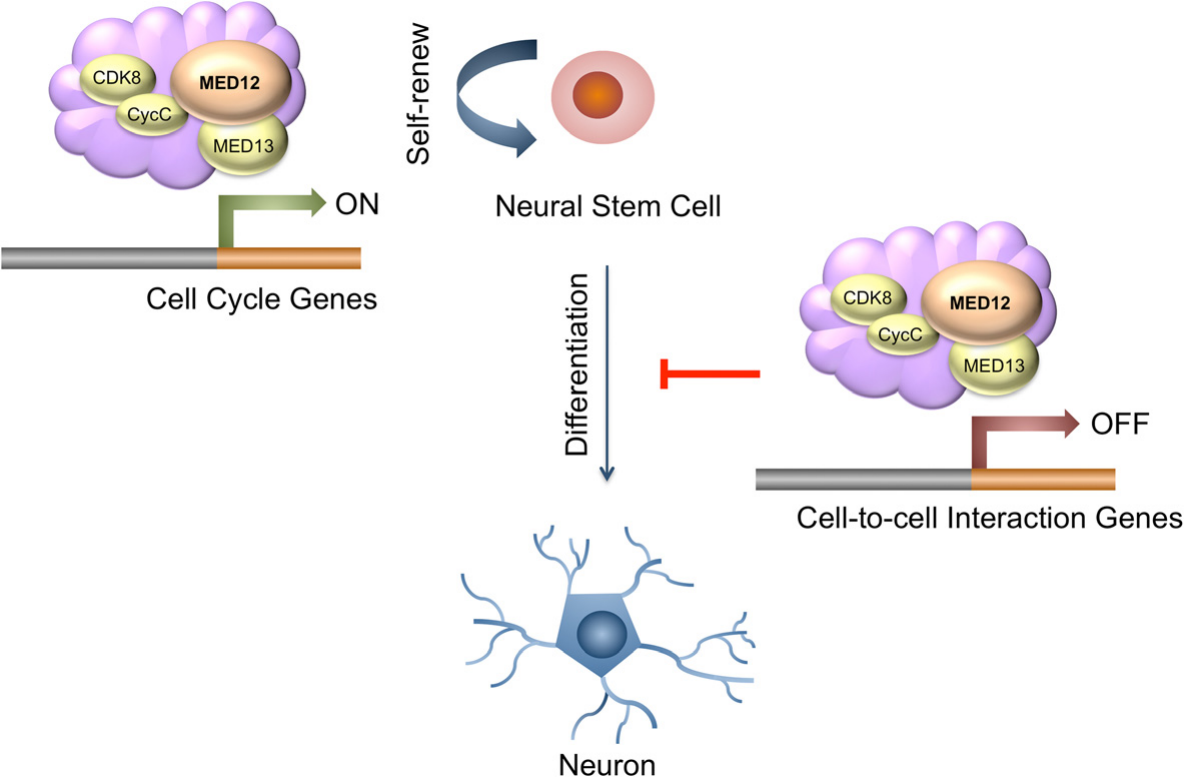
Schematic model for the role of Med12 in mNSCs. Med12 contributes to the maintenance of NSC identity through a functionally bipartite role in suppression and activation of gene expression programs dedicated to cell adhesion and G1/S phase cell cycle progression, respectively

Collectively, our findings reveal new insight concerning the global contribution of Med12 to NSC biology. We propose a model in which Med12 contributes to maintenance of NSC identity by concurrent suppression and activation of gene expression programs dedicated to cell adhesion and G1/S phase cell cycle progression, respectively (Fig. 7). In this capacity, Med12 may contribute to the balance between NSC self-renewal and differentiation. Future studies will seek to clarify the mechanistic bases by which Med12/Mediator integrates and transduces regulatory signals that specify its dual functionality in suppression and activation of gene expression programs that contribute to NSC fate.

## Methods

### Mouse NSC maintenance and neuronal differentiation

mNS-5 cells (a kind gift form Dr. Austin Smith, Wellcome Trust Medical Research Council Stem Cell Institute, University of Cambridge, UK) were cultured as described [41]. Briefly, cells were maintained on 0.1 % (*v*/*v*) gelatin-coated tissue culture plates in RHB-A media (StemCells, Inc., Newark, CA, USA) supplemented with FGF-2 (10 ng/ml; Peprotech, Rocky Hill, New Jersey, USA), EGF (10 ng/ml; Peprotech) and L-glutamine (2 mM; Invitrogen, CA, USA), penicillin (100 units/ml; Invitrogen, CA, USA), streptomycin (100 ug/ml; Invitrogen). The cells were maintained in 5 % CO2 incubator at 37 °C. Accutase (Sigma-Aldrich, St Louis, MO, USA) was used for passaging.

Neuronal differentiation was achieved through withdrawal of growth factors sequentially on laminin substratum. Cells were plated on poly-ornithine/laminin coated tissue culture plate in RHB-A medium supplemented with 5 ng/ml FGF-2. Medium was replaced every 2–3 days during the differentiation. After 7 days, the medium was changed to RHB-Basal medium mixed with Neural Basal Media (1:1) (GIBCO) supplemented with ¼ N2 and B27 (GIBCO) without growth factors.

### Lentivirus-mediated RNAi

shRNA transfer vectors were purchased from Sigma (Sigma; catalog numbers: shControl- SHC002, shMed12- TRCN0000096467, shCdk8- TRCN0000023107, shCyclinC- TRCN0000077830, shMed1- TRCN0000099578, shLamc1- TRCN0000055420, shSdc2- TRCN0000311814, Itgb5- TRCN0000067123). Each lentiviruses were produced by transient cotransfection of HEK293T cells with shRNA transfer vectors with pMD2G and psPAX2 (AddGene) using X-tremeGENE9 (Roche Applied Science). For lentivirus infection, mNS-5 cells were seeded in 60 mm tissue culture plate at a density of 1 × 10^6^ cells per plate. Twenty-four hours later, cells were infected with lentiviruses in the presence of 8 μg/ml of polybrene (Millipore). Twenty-four hours after lentivirus infection, the medium was removed and replaced with fresh medium containing 600 ng/ml puromycin (Gibco) for selection.

### RNA isolation and microarray analysis

Three independent biological replicates of nonsilencing control and Med12-specific shRNA infected cells were prepared for gene expression microarray analysis. Cells were harvested 5.5 days after virus infection and total RNA was isolated using the RNeasy Mini Kit (Qiagen, Valencia, CA) following the manufacturer’s protocol. RNA was quantified using a NanoDrop 2000c spectrophotometer, and RNA quality was assessed using Agilent Bioanalyzer (Agilent Technologies, Inc., Santa Clara, CA). Double-stranded cDNA was synthesized from 500 ng total RNA and subsequently subjected to biotin-labeled cRNA by llumina® TotalPrep RNA Amplification Kit (Ambion, Applied Biosystems, Foster City, CA). The quality of cRNA was assessed using the same way as described above. Biotinylated cRNA was hybridized onto Illumina MouseWG-6 BeadChip (Illumina, San Diego, CA) according to the manufacturer’s protocol.

Arrays were scanned using Illumina iScan System. Raw signal intensities of gene expression data were processed, background subtracted and quantile normalized using Genome Studio (Illumina). Data was exported from Genome Studio and combined with annotation file and experimental parameter file to import into GeneSpring GX (Agilent) for statistical analysis and visualization (including clustering).

Differentially expressed genes (Benjamini-Hochberg corrected *p*-value of *p* ≤ 0.05 and fold change ≥2) were exported for Ingenuity® Pathway analysis (Ingenuity Systems, Mountain View, CA) to identify biological significance of the transcriptional changes observed in Med12 depleted mNS-5 cells.

### Quantitative RT-PCR analysis

Gene expression was measured by quantitative real-time PCR analysis. Briefly, 1 μg of total RNA extracted from infected mNS-5 cells were subjected to reverse-transcription using ImProm-II Reverse Transcription System (Promega) according to the manufacturer’s instruction. Quantitative PCR was carried out using Applied Biosystems 7900HT Fast Real-Time PCR system in 10ul reactions containing 5ul of Absolute SYBR Green ROX mix (Applied Biosystems®, Foster City, CA) and each primer at 70 nM. Primers were synthesized by IDT (Integrative DNA Technologies, Coralville, IA) and tested for specificity and amplification efficiency. The sequences of the primers used are listed in Additional file 4: Table S2. Relative mRNA levels were calculated using the comparative CT method, as described by the manufacturer (Applied Biosystems®), using GAPDH or β-actin Ct values for normalization. Statistical significance was evaluated by Student’s *t*-test.

### Cell adhesion assay

Cells were seeded at a concentration of 1 × 10^6^ per 60 mm tissue culture plate. After 24 h, cells were infected with shRNA-expressing lentivirus for 24 h and subject to puromycin selection as previously described. Six days post-infection, cells were labeled with calcein AM (Life Technologies™), and re-seeded on black-walled 96 well plates. After 2-hour incubation at 37 °C, nonadherent calcein-labeled cells were removed by washing and fluorescence was measured using Fluoroskan Ascent FL (Thermo Scientific). The percentage of adhesion was determined by dividing the corrected (background subtracted) fluorescence of adherent cells by the total corrected fluorescence of cells added to each microplate well and multiplying by 100 %.

For knockdown-differentiation-adhesion assays, cells were seeded at a concentration of 1 × 10^6^ per 60 mm tissue culture plate. After 24 h, cells were infected with shRNA-expressing lentiviruses for 24 h and then subject to puromycin selection. Five days post-infection, cells were re-seeded onto T-75 tissue culture flask and initiated to differentiate toward neuronal lineage. Adhesion assay was performed 40 h following the start of the neuronal differentiation. Brightfield images were obtained by Nikon Eclipse TE300 inverted optical microscope (Nikon) 1, 4, and 7 days after neuronal differentiation initiation.

### Proliferation assay

Cells were seeded at a concentration of 1 × 10^6^ per 60 mm tissue culture plate. After 24 h, cells were infected with NS, Med12-, Cdk8-, Med1- specific shRNA-expressing lentiviruses at 60–70 % confluency and subject to puromycin selection 24 h after virus infection (Gibco, CA, USA). Cells were re-seeded in triplicate on 12-well plates 4 days post-infection, and proliferation was monitored using viable cell counting after trypan blue staining 24, 48, 72, and 96 h after re-seeding. Statistical significance was evaluated by Student’s *t*-test.

### Cell cycle and apoptosis assay

Cells were infected with shRNA-expressing lentivirus at 60–70 % confluency and selected by puromycin 24 h after virus infection (Gibco, CA, USA). Five days post-infection, cells were detached using Accutase (Sigma-Aldrich, St Louis, MO, USA), washed with PBS, and fixed with 1 ml of ice-cold 70 % ethanol. Following centrifugation, cells were washed with PBS and stained with 20 μg/ml PI (Invitrogen) solution in PBS containing 0.1 % Triton X-100 (Sigma-Aldrich, St Louis, MO, USA) and 0.2 mg/ml DNase-free RNase A (Invitrogen, CA, USA) and incubated in the dark at 37 °C for 30 min. Cells were acquired by the FACSCalibur flow cytometer (BD Bioscience, San Jose, CA) with the CellQuest Pro software and then analyzed with the FlowJo software (TreeStar INC, Ashland, OR). PI-Area and PI-Width parameters were used as doublet discriminators and at least 10,000 single cell events were collected.

For apoptosis, cells were stained with annexin V conjugated to APC and propidium iodide (eBioscience, San Diego, CA) according to the manufacturer’s instructions and were acquired on the FACSCalibur within 1 h. The data was analyzed using the FlowJo software.

### Preparation of cell lysate and western blot analysis

Nuclear extracts were prepared in buffer C (20 mM Hepes pH 7.9, 20 % Glycerol, 420 mM NaCl, 1.5 mM MgCl2, 0.2 mM EDTA, 0.1 % NP-40) supplemented with protease inhibitors. Lysates were resolved by 10 %-SDS PAGE, and proteins were analyzed by immunoblot using antibodies against MED12 (Bethyl), CDK8 (Santa Cruz Biotechnology), Cyclin C (BD Biosciences), MED1 (Santa Cruz Biotechnology), MED4 (produced in our laboratory), MED23 (BD Biosciences), MED30 (produced in our laboratory), TFIIHp89 (Santa Cruz Biotechnology), TFIIEβ (Santa Cruz Biotechnology), and TUJ1 (Covance).

### Ethics approval and consent to participate

Not applicable.

### Consent for publication

Not applicable.

### Availability of data and material

The datasets supporting the conclusions of this article are available in the Gene Expression Omnibus (GEO) repository, accession number GSE80624 at http://www.ncbi.nlm.nih.gov/geo/query/acc.cgi?acc=GSE80624.

## Additional files

Additional file 1: Figure S1. Unbiased genome-wide expression profiling analysis validation (A, C, D) RNA from mNS-5 NSCs infected with lentiviruses expressing non-specific (NS) control or Med12-specific shRNAs was harvested and subjected to RT-qPCR analysis. mRNA levels for each gene were normalized to β-actin mRNA and expressed relative to their corresponding mRNA levels in NS control shRNA-expressing cells. In (C, D) relative mRNA levels for 29 randomly selected genes from the original microarray-derived list were determined using the same RNA samples subjected to microarray analysis (C) as well as RNA from an independent set of knockdown experiments (D). Data represent the mean +/− SEM of at least three independent experiments performed in triplicate. Asterisks denote statistically significant differences relative to NS control shRNA (Student’s *t*-test, **p* < 0.1, ***p* < 0.05, ****p* < 0.01). (B) Nuclear extracts from mNS-5 NSCs infected with lentiviruses expressing non-specific (NS) control or Med12-specific shRNAs were harvested and resolved by SDS-10 % PAGE prior to processing by immunoblot using antibodies specific for Med12, Cdk8, the p89 subunit of transcription factor IIH (p89), and the β subunit of transcription factor IIE (TfIIeβ). Both p89 and TfIIeβ served as an internal loading controls. (TIF 16612 kb) Additional file 2: Table S1. Differentially regulated genes by Med12 KD in mNS-5 NSCs. (DOCX 100 kb) Additional file 3: Figure S2. Validation of Med12 and Cdk8 depletion in representative knockdown experiment. Nuclear extracts from mNS-5 NSCs infected with lentiviruses expressing non-specific (NS) control or Med12-specific shRNAs were harvested and resolved by SDS-10 % PAGE prior to processing by immunoblot using antibodies specific for Med12, Cdk8, the p89 subunit of transcription factor IIH (p89), the latter of which served as an internal loading control. (TIF 917 kb) Additional file 4: Table S2. RT-qPCR primer sequences. (DOCX 106 kb)

## Abbreviations

ECM: extracellular matrix
NSC: neural stem cell
PCR: polymerase chain reaction
PIC: pre-initiation complex
Pol II: RNA polymerase II
REST/NRSF: RE1 silencing transcription factor/neural restrictive silencer factor
RNAi: RNA interference
shRNA: short hairpin RNA
XLID: X-linked intellectual disability
