# Supplementary figures and images for: Mediator subunit Med12 contributes to the maintenance of neural stem cell identity
Source: BMC Dev Biol. 2016 May 17;16:17. doi: 10.1186/s12861-016-0114-0 (PMC4869265; doi:10.1186/s12861-016-0114-0)

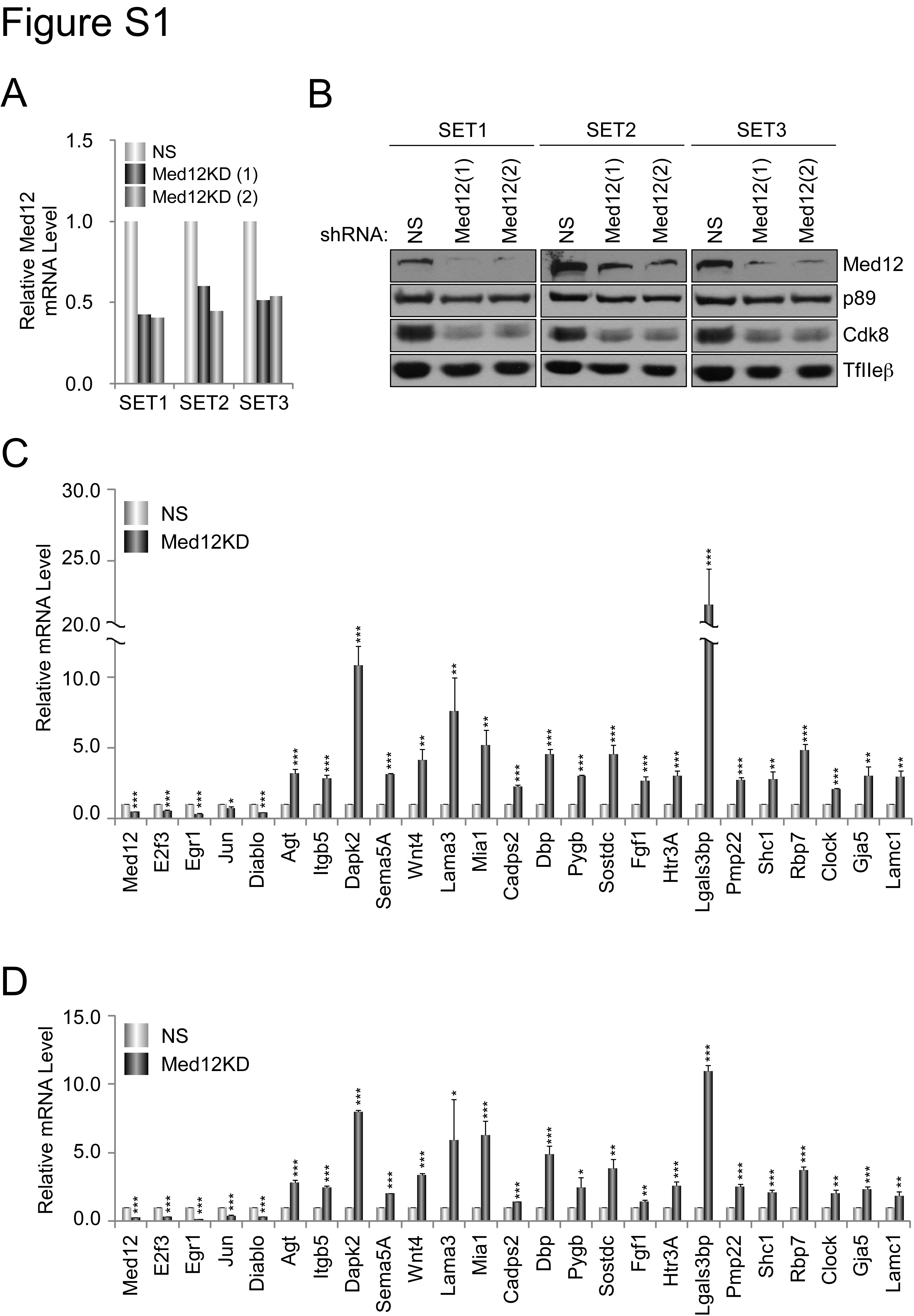

Supplement: Additional file 1: Figure S1. — Unbiased genome-wide expression profiling analysis validation (A, C, D) RNA from mNS-5 NSCs infected with lentiviruses expressing non-specific (NS) control or Med12-specific shRNAs was harvested and subjected to RT-qPCR analysis. mRNA levels for each gene were normalized to β-actin mRNA and expressed relative to their corresponding mRNA levels in NS control shRNA-expressing cells. In (C, D) relative mRNA levels for 29 randomly selected genes from the original microarray-derived list were determined using the same RNA samples subjected to microarray analysis (C) as well as RNA from an independent set of knockdown experiments (D). Data represent the mean +/− SEM of at least three independent experiments performed in triplicate. Asterisks denote statistically significant differences relative to NS control shRNA (Student’s t-test, *p < 0.1, **p < 0.05, ***p < 0.01). (B) Nuclear extracts from mNS-5 NSCs infected with lentiviruses expressing non-specific (NS) control or Med12-specific shRNAs were harvested and resolved by SDS-10 % PAGE prior to processing by immunoblot using antibodies specific for Med12, Cdk8, the p89 subunit of transcription factor IIH (p89), and the β subunit of transcription factor IIE (TfIIeβ). Both p89 and TfIIeβ served as an internal loading controls. (TIF 16612 kb) [file 12861_2016_114_MOESM1_ESM.tif]

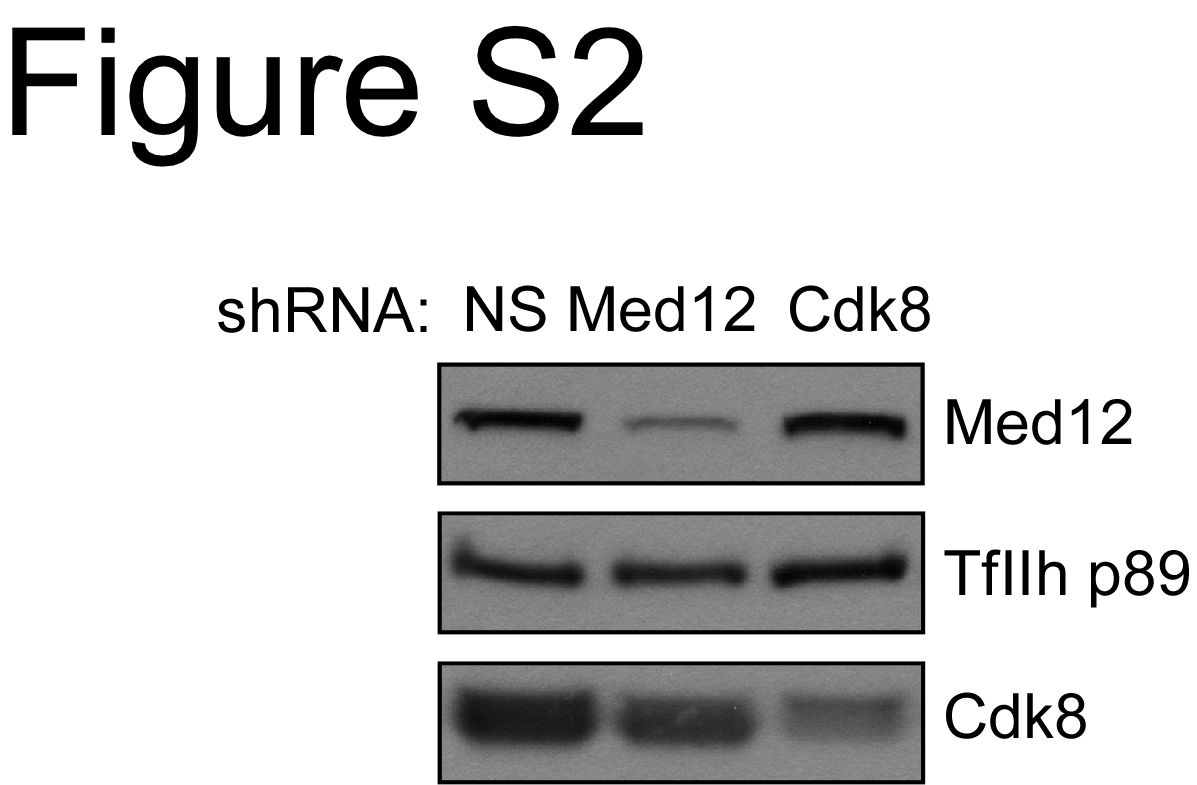

Supplement: Additional file 3: Figure S2. — Validation of Med12 and Cdk8 depletion in representative knockdown experiment. Nuclear extracts from mNS-5 NSCs infected with lentiviruses expressing non-specific (NS) control or Med12-specific shRNAs were harvested and resolved by SDS-10 % PAGE prior to processing by immunoblot using antibodies specific for Med12, Cdk8, the p89 subunit of transcription factor IIH (p89), the latter of which served as an internal loading control. (TIF 917 kb) [file 12861_2016_114_MOESM3_ESM.tif]
