# Supplementary material for: Mediator subunit Med12 contributes to the maintenance of neural stem cell identity
Source: BMC Dev Biol. 2016 May 17;16:17. doi: 10.1186/s12861-016-0114-0 (PMC4869265; doi:10.1186/s12861-016-0114-0)
Supplement: Additional file 2: Table S1. — Differentially regulated genes by Med12 KD in mNS-5 NSCs. (DOCX 100 kb) [file 12861_2016_114_MOESM2_ESM.docx]

## Table S1. Differentially regulated genes by Med12 KD in mNS-5 NSC

| Accession Number | Gene Symbol | Fold Change |
| --- | --- | --- |
| NM_011079.2 | Phkg1 | 152.87 |
| NM_133670.1 | Sult1a1 | 84.37 |
| NM_007621.1 | Cbr2 | 53.86 |
| NM_023887.3 | Gcnt2 | 53.8 |
| NM_007652.2 | Cd59a | 49.9 |
| NM_011150.2 | Lgals3bp | 49.5 |
| NM_175462.3 | Kcnt1 | 40.99 |
| NM_025590.3 | Acot11 | 37.13 |
| NM_181588.3 | Cmbl | 36.39 |
| NM_145551.3 | Slc5a9 | 31.53 |
| NM_029674.1 | Got1l1 | 27.01 |
| NM_146136.1 | Slc16a4 | 25.63 |
| NM_001081285.1 | 620807 | 21.11 |
| NM_025734.2 | Kcng4 | 20.44 |
| NM_008760.2 | Ogn | 19.16 |
| XM_001480162.1 | Fer1l3 | 18.9 |
| NM_011570.2 | Tes | 15.62 |
| NM_029803.1 | Ifi27 | 14.8 |
| NM_001013013.1 | Dhrs7c | 14.4 |
| NM_031386.1 | Tex14 | 13.36 |
| NM_019394.1 | Mia1 | 12.99 |
| NM_022434.1 | Cyp4f14 | 11.39 |
| XM_126649.3 | 1700023F06Rik | 11.19 |
| NM_025956.3 | 1700011H14Rik | 9.96 |
| NM_016885.1 | Emcn | 9 |
| NM_007899.1 | Ecm1 | 8.87 |
| XM_484933.5 | Pcp4l1 | 8.03 |
| NM_138741.1 | Sdpr | 8.02 |
| NM_013883.1 | Scmh1 | 7.36 |
| NM_011271.2 | Rnase1 | 7.26 |
| NM_177584.3 | Btla | 6.91 |
| NM_001033185.2 | 1700084C01Rik | 6.11 |
| NM_010473.2 | Hrc | 5.95 |
| NM_013603.1 | Mt3 | 5.37 |
| NM_019517.3 | Bace2 | 5.29 |
| NM_025312.2 | Sostdc1 | 5.13 |
| NM_001033144.1 | 1190007F08Rik | 5.04 |
| NM_029270.1 | Arhgap24 | 5.02 |
| NM_181728.1 | Art3 | 4.9 |
| AK042789 | A730024G14Rik | 4.86 |
| NM_145562.2 | 9130213B05Rik | 4.81 |
| NM_001033042.3 | AI595366 | 4.76 |
| NM_011578.2 | Tgfbr3 | 4.69 |
| XM_001475611.1 | LOC100046120 | 4.67 |
| NM_025508.3 | Gmpr | 4.63 |
| NM_008288.2 | Hsd11b1 | 4.51 |
| NM_016758.2 | Rgs14 | 4.49 |
| NM_008121.2 | Gja5 | 4.48 |
| NM_007586.1 | Calb2 | 4.43 |
| NM_173391.1 | Tph2 | 4.4 |
| NM_010019.2 | Dapk2 | 4.34 |
| NM_008183.3 | Gstm2 | 4.21 |
| NM_138304.1 | Calml4 | 4.21 |
| XM_128926.3 | Lama3 | 4.06 |
| NM_133775.1 | Il33 | 3.93 |
| NM_177756.3 | Glt25d2 | 3.92 |
| NM_178894.2 | AA792892 | 3.91 |
| XM_142538.2 | LOC235966 | 3.87 |
| NM_009154.1 | Sema5a | 3.76 |
| NM_001033240.1 | Wfdc6a | 3.73 |
| NM_009155.3 | Sepp1 | 3.63 |
| XM_354697.3 | A230065H16Rik | 3.62 |
| NM_011584.3 | Nr1d2 | 3.57 |
| NM_022020.2 | Rbp7 | 3.57 |
| NM_007417.2 | Adra2a | 3.57 |
| NM_146174.1 | Fam115c | 3.55 |
| NM_181348.4 | 6330414G02Rik | 3.54 |
| NM_009523.1 | Wnt4 | 3.52 |
| NM_054077.3 | Prelp | 3.47 |
| NM_175138.3 | Dnaic1 | 3.46 |
| NM_027857.3 | Acy3 | 3.44 |
| NM_007428.3 | Agt | 3.36 |
| NM_172285.1 | Plcg2 | 3.35 |
| NM_201641.2 | Ugt1a10 | 3.34 |
| NM_026725.2 | Dusp23 | 3.32 |
| NM_016974.1 | Dbp | 3.3 |
| NM_054042.2 | Cd248 | 3.27 |
| XM_895387.2 | 2210408F21Rik | 3.16 |
| NM_007752.2 | Cp | 3.12 |
| NM_001163610.1 | 1110062M06Rik | 3.11 |
| NM_032003.1 | Enpp5 | 2.99 |
| NM_172161.2 | Irak2 | 2.96 |
| NM_177030.3 | Dock6 | 2.96 |
| NM_172520.2 | Arhgef19 | 2.92 |
| NM_175003.3 | AU040829 | 2.88 |
|  | 1110059G02Rik | 2.87 |
| NM_021351.1 | Cryba4 | 2.87 |
| NM_172710.3 | 2310045A20Rik | 2.83 |
| XM_001475483.1 | LOC100046056 | 2.82 |
| NM_029631.2 | Abhd14b | 2.81 |
| NM_177469.3 | Gpr123 | 2.78 |
| NM_153781.1 | Pygb | 2.75 |
| NM_019877.2 | Copz2 | 2.71 |
| NM_028995.3 | Npal3 | 2.71 |
| NM_025659 | Abi3 | 2.71 |
| NM_027988.1 | Noxo1 | 2.65 |
| NM_025378.2 | Ifitm3 | 2.64 |
| NM_007495.2 | Astn1 | 2.62 |
| XM_620286.3 | Samd9l | 2.6 |
| NM_134127.1 | Cyp4f15 | 2.58 |
| NM_011656.2 | Tuft1 | 2.57 |
| NM_026376.3 | Plxnd1 | 2.57 |
| NM_153513.1 | BC028528 | 2.57 |
| NM_172768 | A930008A22Rik | 2.56 |
| NM_008304.2 | Sdc2 | 2.55 |
| NM_010580.1 | Itgb5 | 2.55 |
| NM_133221.1 | Slc24a6 | 2.54 |
|  | D1Ertd471e | 2.52 |
| NM_207269.1 | D330050I23Rik | 2.51 |
| NM_010683.2 | Lamc1 | 2.51 |
| NM_027868.2 | Slc41a3 | 2.5 |
| NM_176941.2 | A330041J22Rik | 2.49 |
| NM_008182.3 | Gsta2 | 2.48 |
| NM_013415.5 | Atp1b2 | 2.48 |
| NM_023245.3 | Palmd | 2.47 |
| XR_002313.1 | 5830431A10Rik | 2.46 |
| NM_001099644.1 | Htr3a | 2.44 |
| NM_027032.2 | Pacrg | 2.43 |
| NM_021384.3 | Rsad2 | 2.43 |
| NM_009242.1 | Sparc | 2.42 |
| NM_008855.2 | Prkcb | 2.42 |
| NM_153163.3 | Cadps2 | 2.4 |
| NM_011180.2 | Pscd1 | 2.4 |
| NM_001081066.1 | Dennd3 | 2.4 |
| NM_001001326.1 | St5 | 2.38 |
| NM_013534.4 | Leprel2 | 2.38 |
| NM_001085515.1 | AI464131 | 2.38 |
| NM_009384.2 | Tiam1 | 2.35 |
| NM_025988.2 | Acbd4 | 2.35 |
| NM_153584.1 | BC031353 | 2.33 |
| NM_028841.1 | Tspan17 | 2.33 |
| XM_921606.2 | 2610307O08Rik | 2.33 |
| NM_178111.3 | Trp53inp2 | 2.32 |
| NM_025836.3 | M6prbp1 | 2.32 |
| AK017479 | Cgef2-pending | 2.31 |
| NM_009287.4 | Stim1 | 2.29 |
| NM_139300.3 | Mylk | 2.28 |
| NM_010197.3 | Fgf1 | 2.27 |
| NM_177776.3 | Smtnl2 | 2.27 |
| NM_198607.1 | 4930572J05Rik | 2.27 |
| NM_027288.2 | Manba | 2.26 |
|  | 2310033F14Rik | 2.25 |
| NM_029186.2 | Tmem180 | 2.25 |
| NM_001001980.2 | Limch1 | 2.24 |
| NM_026866.2 | Disp1 | 2.23 |
| NM_010189 | Fcgrt | 2.23 |
| NM_138744.2 | Ssx2ip | 2.22 |
| NM_001033226.2 | Calr4 | 2.21 |
| NM_030700.1 | Maged2 | 2.2 |
| NM_011175.2 | Lgmn | 2.2 |
| NM_009932.2 | Col4a2 | 2.19 |
| NM_008592.2 | Foxc1 | 2.19 |
| NM_133943.2 | Hsd3b7 | 2.19 |
| NM_010656.2 | Sspn | 2.18 |
| NM_010517.2 | Igfbp4 | 2.18 |
| NM_023908.2 | Slco3a1 | 2.17 |
| NM_172543.2 | 5730593F17Rik | 2.17 |
| NM_145512 | 2010005O13Rik | 2.17 |
| NM_008885.2 | Pmp22 | 2.16 |
| NM_181072 | Myo1e | 2.16 |
| NM_021551.3 | Slc22a17 | 2.16 |
| NM_175503.3 | Aard | 2.15 |
| NM_013869.3 | Tnfrsf19 | 2.14 |
| NM_145516.2 | Plekhb2 | 2.14 |
| NM_007715.5 | Clock | 2.09 |
| NM_172845.1 | Adamts4 | 2.08 |
| NM_001083587.1 | Tns3 | 2.08 |
| NM_029640.1 | Trappc9 | 2.07 |
| NM_133778.2 | Fam131a | 2.07 |
| NM_009730.2 | Atrn | 2.06 |
| NM_013850.1 | Abca7 | 2.02 |
| NM_026739.1 | 9530077C05Rik | 2.02 |
| NM_028235.1 | Ttc30b | 2.01 |
| NM_153785.3 | Cdkl3 | 2.01 |
| NM_172827.3 | 2010309L07Rik | 2.01 |
| NM_021487.1 | Kcne1l | 2.01 |
| NM_212486.2 | Gimap8 | 2 |
| XR_034455.1 | LOC100048372 | -2 |
| NM_145465.1 | Stk24 | -2.02 |
| NM_008667.2 | Nab1 | -2.03 |
| NM_022019.4 | Dusp10 | -2.03 |
| NM_023910.5 | Tsc22d4 | -2.03 |
| NM_001177845.1 | C130027E04Rik | -2.04 |
| NM_010591.1 | Jun | -2.05 |
| XM_620647.3 | Gm1815 | -2.06 |
| NM_009272.4 | Srm | -2.07 |
| NM_175439 | C730026E21Rik | -2.07 |
| NM_031877.2 | Wasf1 | -2.07 |
| NM_016714.2 | Nup50 | -2.09 |
| NM_021789.2 | Trappc4 | -2.09 |
| NM_183301.1 | E2f2 | -2.12 |
| NM_009704.3 | Areg | -2.12 |
| NM_028023.3 | Cdca4 | -2.13 |
| NM_026967.3 | Rhebl1 | -2.13 |
| NM_001037134.1 | Ccne2 | -2.14 |
| NM_001007573.1 | Gm50 | -2.18 |
| NM_144874.3 | Cox15 | -2.19 |
| NM_023166.1 | Mtvr2 | -2.21 |
| NM_013664.2 | Sh3gl1 | -2.21 |
| AK054037 | Ankra2 | -2.21 |
| NM_001036293.2 | Nrbf2 | -2.23 |
| NM_010093.2 | E2f3 | -2.24 |
| NM_054096.1 | Tirap | -2.27 |
| NM_172938.2 | Scml4 | -2.28 |
| NM_021366.3 | 9430029L20Rik | -2.29 |
| NM_027773.1 | 2310047D13Rik | -2.32 |
| NM_007475.4 | Rplp0 | -2.33 |
| XM_001481319.1 | Rnf165 | -2.34 |
| NM_009237.1 | Sox3 | -2.38 |
| NM_177561.3 | 1190009E20Rik | -2.41 |
| NM_030081.2 | Zfyve20 | -2.48 |
| NM_080634.3 | Hps3 | -2.5 |
| NM_175185.3 | Hsdl1 | -2.5 |
| NM_016660.2 | Hmga1 | -2.53 |
| NM_024270.2 | Stard3nl | -2.53 |
| NM_026788.1 | 1110019K23Rik | -2.6 |
| NM_007913.5 | Egr1 | -2.64 |
| NM_173430.2 | Fkrp | -2.66 |
| NM_007589.4 | Calm2 | -2.66 |
| NM_145525.2 | Osbpl6 | -2.71 |
| NM_027326.3 | Mllt3 | -2.73 |
| XM_001475710.1 | LOC100046163 | -2.73 |
| NM_009786.1 | Cacybp | -2.76 |
|  | A630034I12Rik | -2.99 |
| NM_016916.3 | Blcap | -3.07 |
| XM_205232.5 | Gm715 | -3.08 |
| NM_016861.3 | Pdlim1 | -3.1 |
| NM_026091.2 | 1700037H04Rik | -3.15 |
| NM_138596.1 | Med10 | -3.19 |
| NM_026864.1 | Rasl11a | -3.36 |
| NM_028603.1 | 2410081M15Rik | -3.44 |
| NM_153420.2 | Acpl2 | -3.48 |
| NM_023232.3 | Diablo | -3.49 |
| NM_001001880.2 | Mpzl1 | -3.56 |
| NM_026854.2 | Dtwd2 | -3.6 |
| NM_023162.4 | Znrd1 | -3.68 |
| NM_016803.2 | Chst3 | -4.46 |
| NM_053103.5 | 1810020C02Rik | -4.47 |
| NM_031256.2 | Plekha3 | -5.25 |
| XM_149592.1 | 5031425E22Rik | -6.19 |
