# Supplementary material for: Mediator subunit Med12 contributes to the maintenance of neural stem cell identity
Source: BMC Dev Biol. 2016 May 17;16:17. doi: 10.1186/s12861-016-0114-0 (PMC4869265; doi:10.1186/s12861-016-0114-0)
Supplement: Additional file 4: Table S2. — RT-qPCR primer sequences. (DOCX 106 kb) [file 12861_2016_114_MOESM4_ESM.docx]

## Table S2. Primer sequences for RT-qPCR

| Gene Symbol | Strand | Sequence (5' 🡪 3') |
| --- | --- | --- |
| β-actin | Forward | TGCACCACCAACTGCTTAG |
|  | Reverse | GGATGCAGGGATGATGTTC |
| Gapdh | Forward | GATCATTGCTCCTCCTGAGC |
|  | Reverse | GAAAGGGTGTAAAACGCAGC |
| Med12 | Forward | TTGAGCCAACAGCCTTTCTT |
|  | Reverse | TCATGCATTAGCTGCTTTGG |
| Cdk8 | Forward | AGCTTCGAAAGCCAACAAGAAGCC |
|  | Reverse | GCAACACCCAGTTGGCATGAAGAT |
| E2f3 | Forward | TGCTGGAAGGCATCCACCTCATTA |
|  | Reverse | AGTTTGAGGTCCAGGGTACAGCTT |
| Egr1 | Forward | AACAACCCTATGAGCACCTGACCA |
|  | Reverse | ACAAAGTGTTGCCACTGTTGGGTG |
| Jun | Forward | GAACTGCATAGCCAGAACACGCTT |
|  | Reverse | TGAAGTTGCTGAGGTTGGCGTAGA |
| Diablo | Forward | TGTGTGCGGTTCCTATTGCTCAGA |
|  | Reverse | ATTGCTTCAATCAGCGCGTAGGTG |
| Agt | Forward | TTCACTGCTCCAGGCTTTCGTCTA |
|  | Reverse | ATCTTCCACCCTGTCACAGCCTTT |
| Itgb5 | Forward | TGTTCAGCTACACAGAACTGCCCA |
|  | Reverse | TTTGGAACTTGGCAAACTCTCGGC |
| Dapk2 | Forward | TGTGGAGCATTGGAGTCATCACCT |
|  | Reverse | TAAGCCGTTTCCGGGTCTCTTTCA |
| Sema5a | Forward | GGCAGCTGTTTGCTGGAAGAGATT |
|  | Reverse | ATCTTGGCCACATGCTCCTGTAGT |
| Wnt4 | Forward | ATGCAGCAGTGGAGAACTGGAGAA |
|  | Reverse | TCCACAAAGGACTGTGAGAAGGCT |
| Lama3 | Forward | ATGGTCAGGGAAGCCAACGGAATA |
|  | Reverse | AGTTATTGGCATCGACCAGAGCCT |
| Mia1 | Forward | CTTGGCATCGTCGTCTTGTCTGTT |
|  | Reverse | GGCCCTTCAACTTGGAGAAGACAT |
| Cadps2 | Forward | TGGCTCAACAACTTCCGAGGATCT |
|  | Reverse | CTGGGATGCGCAAGTCAGTTGTTT |
| Dbp | Forward | ACCGTGGAGGTGCTAATGACCTTT |
|  | Reverse | TCTCGACCTCTTGGCTGCTTCATT |
| Pygb | Forward | AAGAAGCTGATGACTGGCTACGCT |
|  | Reverse | AGAGCCTCATGGTGTTGACTGTGT |
| Sostdc1 | Forward | ATGGAACAAAGTACTGGAGCCGGA |
|  | Reverse | CACGCTTTCAAAGTTGTGGCTGGA |
| Fgf-1 | Forward | ATGTCTGTTCCTGGAAAGGCTGGA |
|  | Reverse | GATGGCTTTCTGGCCATAGTGAGT |
| Htr3a | Forward | ACCGGAAGAAGTGAGGTCTGACAA |
|  | Reverse | AGCAAGAGGCTGACTGCGTAGAAT |
| Lgala3bp | Forward | AGCAGCGTCATCATGAGAGTGGAT |
|  | Reverse | AAAGCCGTCCACAGTAGTCCTGAA |
| Pmp22 | Forward | AGTGTTGCTCTTCGTCTCCACCAT |
|  | Reverse | TTCGCTCACTGATGAGGAGTAGCA |
| Shc1 | Forward | GCTGCATCCCAACGACAAAGTCAT |
|  | Reverse | GCACAGCTTCACACACCAAACTGA |
| Rbp7 | Forward | TGAGGAGGATAACAAAGGCCTGGA |
|  | Reverse | ATCAGGCTCTCTGGAAGGTTTGCT |
| Clock | Forward | TGCTTCCTGGTAACGCGAGAAAGA |
|  | Reverse | AGGAATGTGGGTTTCCAGTCCTGT |
| Gja5 | Forward | GAAGTGCCAAACCAGGAGCAGATT |
|  | Reverse | AAGGCGCCGTTTGTCACTATGGTA |
| Lamc1 | Forward | TGAGGTGAATGGTATGCTGAGGCA |
|  | Reverse | TGAGCTTGTTCAGGTCCACTGTGT |
| Sdc2 | Forward | TGGTGGTGTGATCGGCTTTCTCTT |
|  | Reverse | TGGTTTGCGTTCTCCAAGGTCGTA |
| Sparc | Forward | TGTTGGCCCGAGACTTTGAGAAGA |
|  | Reverse | ACCCATCAATAGGGTGCTGATCCA |
| Tuj1 | Forward | ACACCTATTCAGGCCCGACAACTT |
|  | Reverse | TCTCACACTCTTTCCGCACGACAT |
| Ccnc | Forward | AGACCTTTGCTCCAGTATGTGCAG |
|  | Reverse | AAGCGATCATGAACGGAGGGTACA |
| Med1 | Forward | GAGCATCAAAGCCAAAGTGACGCT |
|  | Reverse | AGCCTGACTCACTTTCACTGTCCA |
| Ccnd1 | Forward | TGCTGCAAATGGAACTGCTTCTGG |
|  | Reverse | TACCATGGAGGGTGGGTTGGAAAT |
| Ccnd2 | Forward | TGCAGAAGGACATCCAACCGTACA |
|  | Reverse | AAGCTAGGAACATGCACACTGCAC |
| Ccnd3 | Forward | TGGCTATGAACTACCTGGATCGCT |
|  | Reverse | ACAGCCTGGTCCGTATAGATGCAA |
| E2f1 | Forward | GCATCCAGCTCATTGCCAAGAAGT |
|  | Reverse | GAAAGCAGTTGCAGCTGTGTGGTA |
| E2f2 | Forward | AGCACCTGACCGAAGATAATGCCA |
|  | Reverse | TGTCCGGCACTTCCAATCTTGTCT |
| E2f3 | Forward | TGCTGGAAGGCATCCACCTCATTA |
|  | Reverse | AGTTTGAGGTCCAGGGTACAGCTT |
| E2f4 | Forward | TGGTAGCCCTGGAACTGAGAACAA |
|  | Reverse | TGCTGCTGCTGCTGCTACTACTAT |
| Ccne1 | Forward | CCTCCAAAGTTGCACCAGTTTGCT |
|  | Reverse | TCGTTGACATAGGCCACTTGGACA |
| Ccne2 | Forward | TCTGGAGGAATCAGCCCTTGCATT |
|  | Reverse | TTGCCAAACCTCCTGTGAACATGC |
| Ccna2 | Forward | TACCTGCCTTCACTCATTGCTGGA |
|  | Reverse | ATTGACTGTTGGGCATGTTGTGGC |
| p21 | Forward | AATCCTGGTGATGTCCGACCTGTT |
|  | Reverse | GTGACGAAGTCAAAGTTCCACCGT |
| p27 | Forward | AGAAATCTCTTCGGCCCGGTCAAT |
|  | Reverse | GTCGAAATTCCACTTGCGCTGACT |
